# 2881. Effect of Antimicrobial Stewardship on Linezolid Use and Resistance in *Enterococcus*: a Quasi-Experimental Difference-in-Differences Study

**DOI:** 10.1093/ofid/ofad500.158

**Published:** 2023-11-27

**Authors:** Edward Kong, Erica S Shenoy, Alyssa R Letourneau

**Affiliations:** Harvard Medical School & Harvard University, Boston, MA; Massachusetts General Hospital, Boston, Massachusetts; Massachusetts General Hospital, Boston, Massachusetts

## Abstract

**Background:**

Antimicrobial stewardship programs (ASPs) require significant effort and resources. While a large literature has linked ASP policies to lower antibiotic utilization and costs, ASPs’ effect on antibiotic resistance is less well understood. Most prior studies on resistance have relied on before-vs-after comparisons in a single institution which may be confounded by time trends in resistance. We use a difference-in-differences research design to investigate the impact of hospital-specific ASP policy changes on linezolid-resistant *Enterococcus* (LRE). While LRE has typically been described in case reports or case series, large healthcare-based outbreaks have been reported.

**Methods:**

We used electronic health records data from two large academic hospitals between 2008—2019, during which prevalence of LRE, defined as *Enterococcus* isolates reported as resistant, ranged from 0.3%--2.8%. We identified two ASP policies implemented in Hospital 1 but not Hospital 2: a new ASP approval pager in October 2011 and a linezolid-specific policy that defined appropriate indications and increased pre- and post-prescriptive review in March 2014. We estimated the combined effect of the two ASP policies by comparing changes in linezolid use and LRE in Hospital 1 to changes in Hospital 2, which had no changes in policies related to linezolid during the study period.

**Results:**

Our sample included 521,529 inpatient admissions and 31,440 *Enterococcus* isolates. In Hospital 1, linezolid use dropped sharply after each policy was implemented, relative to the Hospital 2 (Figure 1). The combined policies led to a 22% decrease in linezolid utilization (P < 0.001) relative to the pre-policy mean. Other analyses of approval pager data showed a decline in both the linezolid approval rate and requests made. As the interventions were in effect for inpatient populations only, no effect was observed on outpatient linezolid use.

A significant decrease of 1.4% in LRE was observed for Hospital 1 but not 2 (Figure 2), a 59% decrease relative to the pre-policy mean of 2.4% (P < 0.001).

Effect of ASP policies on linezolid use

**Figure:**
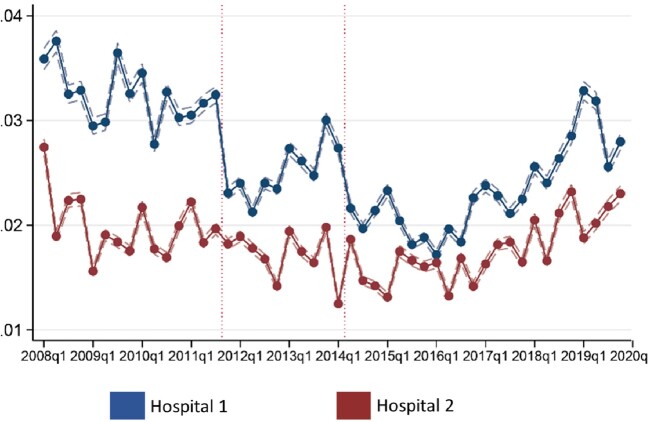

Notes: Figure shows the share of inpatient admissions associated with a linezolid medication administration over time, separately for Hospital 1 (blue line) and Hospital 2 (red line). Data are plotted at the quarterly level. Dashed lines reflect standard errors. The dotted vertical lines indicate the timing of the first and second ASP policies respectively.

Effect of ASP policies on linezolid resistance for Enterococcus isolates

**Figure:**
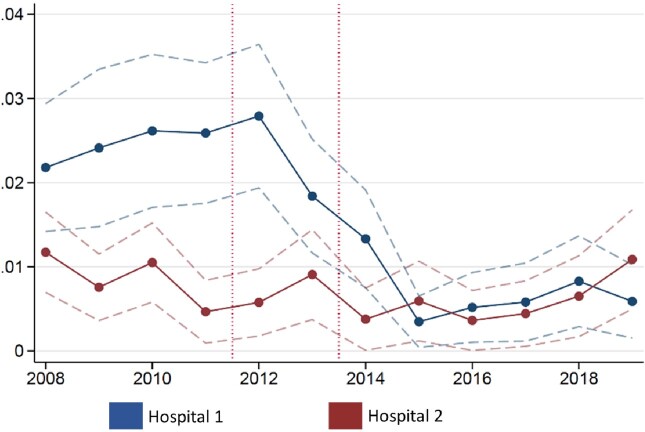

Notes: Figure shows the share of Enterococcus isolates testing “R” to linezolid over time, separately for Hospital 1 (blue line) and Hospital 2 (red line). Data are plotted at the yearly level. Dashed lines reflect standard errors, clustered at the patient level. The dotted vertical lines indicate the timing of the first and second ASP policies respectively.

**Conclusion:**

Using a quasi-experimental research design, we demonstrated that specific ASP interventions were associated with a decrease in linezolid utilization and resistance in *Enterococcus*.

**Disclosures:**

**All Authors**: No reported disclosures

